# Suppression of UVB-Induced MMP-1 Expression in Human Skin Fibroblasts Using Lysate of *Lactobacillus iners* Derived from Korean Women’s Skin in Their Twenties

**DOI:** 10.3390/cimb46010033

**Published:** 2024-01-04

**Authors:** Jin-Sung Lee, Jin-Woo Min, Seong-Bong Gye, Yong-Woo Kim, Hee-Cheol Kang, Yoon-Seo Choi, Won-Sang Seo, Bun-Yeoul Lee

**Affiliations:** 1 Department of Applied Biotechnology, Ajou University, Suwon 16499, Republic of Korea; jsrnd@ajou.ac.kr; 2 R&D Complex, Kolmar Korea, 61, 8-gil, Heolleung-ro, Seocho-gu, Seoul 06800, Republic of Korea; a0075@kolmar.co.kr (S.-B.G.); ywkim@kolmar.co.kr (Y.-W.K.); 3 Green & Biome Customizing Laboratory, GFC Co., Ltd., Hwaseong 18471, Republic of Korea; jw.min@gfcos.co.kr (J.-W.M.); michael@gfcos.co.kr (H.-C.K.); 4 Graduate School-Interdisciplinary Program in Biocosmetics, Sungkyunkwan University, Suwon 16419, Republic of Korea; eveelf@g.skku.edu; 5 Department of Molecular Science and Technology, Ajou University, Suwon 16499, Republic of Korea

**Keywords:** *Lactobacillus*, human skin derived probiotics, UVB-induced, MMP-1, 16S rRNA, ERK, JNK, p38

## Abstract

The process of skin aging is intricate, involving intrinsic aging, influenced by internal factors, and extrinsic aging, mainly caused by exposure to UV radiation, resulting in photoaging. Photoaging manifests as skin issues such as wrinkles and discoloration. The skin microbiome, a diverse community of microorganisms on the skin’s surface, plays a crucial role in skin protection and can be affected by factors like humidity and pH. Probiotics, beneficial microorganisms, have been investigated for their potential to enhance skin health by regulating the skin microbiome. This can be accomplished through oral probiotics, impacting the gut–skin axis, or topical applications introducing live bacteria to the skin. Probiotics mitigate oxidative stress, suppress inflammation, and maintain the skin’s extracellular matrix, ultimately averting skin aging. However, research on probiotics derived from human skin is limited, and there is no established product for preventing photoaging. The mechanism by which probiotics shield the skin microbiome and skin layers from UV radiation remains unclear. Recently, researchers have discovered *Lactobacillus* in the skin, with reports indicating a decrease in this microorganism with age. In a recent study, scientists isolated *Lactobacillus iners* KOLBM20 from the skin of individuals in their twenties and confirmed its effectiveness. A comparative analysis of genetic sequences revealed that strain KOLBM20 belongs to the *Lactobacillus* genus and closely relates to *L. iners* DSM13335(T) with a 99.20% similarity. Importantly, *Lactobacillus iners* KOLBM20 displayed anti-wrinkle properties by inhibiting MMP-1. This investigation demonstrated the inhibitory effect of KOLBM20 strain lysate on MMP-1 expression. Moreover, the data suggest that KOLBM20 strain lysate may prevent UVB-induced MMP-1 expression by inhibiting the activation of the ERK, JNK, and p38 signaling pathways induced by UVB. Consequently, KOLBM20 strain lysate holds promise as a potential therapeutic agent for preventing and treating skin photoaging.

## 1. Introduction

The aging of the skin is a multifaceted process encompassing both structural and functional alterations. Intrinsic aging is guided by internal factors, notably involving inflammatory mediators, while extrinsic aging, like photoaging, is predominantly triggered by exposure to ultraviolet (UV) radiation [1]. Prolonged exposure to ultraviolet radiation (UVR) leads to a condition known as photoaging, characterized by premature skin aging due to UVR exposure [2,3,4]. Skin disorders that occur as a result of aging, mainly due to exposure to sunlight (photoaging), are characterized by symptoms such as wrinkles, discoloration, telangiectasias (spider veins), and a dry, rough skin texture [5,6,7]. The clinical signs of photoaging are a consequence of pathological changes in both the epidermal and dermal cells and tissues. Wrinkles, a notable sign of photoaging, develop because of a decline in dermal fibroblasts, diminished collagen and elastin production, and accelerated degradation of these essential components [8]. Skin photoaging not only impacts aesthetic appearance but also weakens the skin’s natural barrier, elevating the risk of inflammatory skin conditions and even skin malignancies [9].

Recently, experimental differences in skin conditions based on race, age, gender, and other factors have been attributed to the skin microbiome, also known as the third layer of the skin [10]. The skin microbiome has been reported to play a role in protecting the skin from harmful factors and has helped in interpreting statistical differences. The diversity of skin-resident microorganisms that make up the skin microbiome varies depending on external environmental factors such as humidity, temperature, pH, lipid content, and sebum levels [11]. The human epidermis constitutes a dynamic ecosystem that hosts a diverse array of microorganisms thriving in various niches. These microbes engage in a mutually beneficial relationship with their human hosts [12]. The skin encompasses a multitude of habitats, including invaginations and specialized niches, fostering the growth of a wide spectrum of microorganisms. Among its vital functions, the skin serves as a physical barrier, safeguarding against the intrusion of external pathogens and toxic substances. The skin offers a diverse array of habitats influenced by distinct physical and biological factors. The intricate and delicate interaction between the human body and its symbiotic microorganisms defines the balance of this relationship. A disruption of this equilibrium can lead to skin issues [13].

Probiotics refer to microorganisms that provide benefits to the human body, with many of them belonging to the *Lactobacillus* genus. Probiotics are well-known for their positive effects on human health through various mechanisms [14]. Probiotics, active microorganisms with beneficial effects on the host, can modulate the composition of the microbiota in a specific part of the host’s flora [15]. There are limited approaches leveraging the skin microbiome to promote skin health. One of them is the gut–skin axis theory, which suggests that regulating the gut microbiome can influence the balance of the skin microbiome [16]. Growing evidence indicates a connection between oral probiotics and their ability to regulate skin photoaging. Oral probiotics have been found to positively impact the interaction between the gut and skin microbiota, resulting in reduced oxidative stress, suppressed inflammation, maintained immune balance, and the prevention of changes in the skin’s extracellular matrix [17,18,19].

Another approach involves the direct application of particular strains or elements that directly influence the overall skin microbiota on the skin, known as the “topical” approach. This method is gaining greater recognition due to its intuitive nature. Topical applications of probiotics have received extensive research attention as a means to directly modify the skin microbiome, with the goal of preventing and addressing skin photoaging [18]. This approach involves introducing carefully cultured live bacteria to the skin in specific quantities. The objective is to re-establish a balanced skin microbiota and restore immune balance, a concept first proposed as a remedy for skin conditions like acne and seborrhea [20,21].

However, investigations into the influence of probiotics on skin microflora and the subsequent effects on the skin are notably scarce. Probiotics can modulate the microbiome by inhibiting harmful microbes while promoting beneficial ones [22]. Probiotics have several beneficial effects on the skin and can help reduce the signs of skin aging caused by exposure to sunlight. Firstly, probiotics reduce oxidative stress levels, which are closely associated with the development of skin aging [17,23]. They enhance the activity of antioxidant enzymes, decrease the production of reactive oxygen species (ROS), and inhibit signaling pathways involved in the breakdown of collagen and the synthesis of matrix metalloproteinases (MMPs) [23,24]. This ultimately leads to a decrease in the damage to the skin caused by ROS and the aging process. Probiotics have an inhibitory effect on the inflammatory cascade, which is responsible for disrupting the skin barrier, increasing water loss through the skin, and accelerating the aging process. Probiotics can suppress the production of pro-inflammatory cytokines, regulate immune responses, and maintain immune balance [23,25]. They also help alleviate skin inflammation caused by exposure to ultraviolet (UV) radiation, thereby preventing skin aging [26]. Probiotics inhibit the remodeling of the extracellular matrix (ECM), which is a critical process affected by UV exposure [17,18]. They regulate the expressions of MMPs and TIMPs (tissue inhibitors of metalloproteinases), thereby reducing the degradation of collagen and elastin. This results in improved skin structure and elasticity, preventing issues such as roughness, sagging, and wrinkling [18].

Topical probiotics are being studied for their potential benefits in skincare, specifically in addressing photoaging and skin aging [27]. Research suggests that they can help slow down the aging process, reduce oxidative stress, and improve the skin’s barrier function [28]. Studies have explored the use of probiotics fermented with plant extracts and *Nitrosomonas eutropha* to treat wrinkles and improve hyperpigmentation, showing promising results [29,30]. However, there are not many cases where the effectiveness of probiotics alone has been confirmed.

Additionally, it is worth emphasizing that most probiotics currently under investigation do not have their origins in human skin. As the skin goes through the aging process, it experiences not only a reduction in collagen and elastin but also, according to recent research, a decline in Lactobacillus on the skin’s surface as age advances [31]. While there is some research on common *Lactobacillus* strains, the study of *Lactobacillus* isolated from human skin is quite limited, including research involving some of the authors of this study [32].

Apart from the scarce research on probiotics derived from human skin, it is crucial to consider the ongoing efforts to develop probiotic-based skin products aimed at preventing photoaging. Despite these endeavors, a definitive product has yet to be established. This could be attributed to the uncertainty surrounding the theory that explains how probiotics absorb and counteract UV radiation to directly protect the skin microbiome and the underlying skin layers [33].

In summary, this research aims to determine whether probiotics derived from human skin can provide protection against UV radiation. This study intends to verify their protective capabilities against UVB radiation and explore their potential use in preventing UVB-induced skin aging by assessing skin toxicity, anti-inflammatory properties, and UVB protection. Additionally, this study aims to uncover the mechanism behind how Lactobacillus lysate from human skin affects UVB-induced skin wrinkling. This involves investigating the inhibitory effects of Lactobacillus lysate on the expressions of MMP-1 and mitogen-activated protein kinase (MAPK) induced by UVB.

## 2. Materials and Methods

### 2.1. Sample Collection

All procedures conducted in this study adhered to the principles outlined in the Declaration of Helsinki and were duly approved by the Institutional Review Board of the Korea Dermatology Research Institute (KDRI-IRB-201028). Samples were obtained by gently rubbing gauze against the facial skin of individuals in their twenties. These gauze suspensions were prepared by adding distilled water and agitating the mixture, followed by the spreading of 100 μL of each sample on MRS agar plates under aerobic conditions at 37 °C. Single colonies were meticulously isolated and transferred to new MRS agar plates for purification. Strain KOLBM20 was subsequently preserved in MRS broth, enriched with 30% glycerol, and stored at −70 °C.

### 2.2. 16S rRNA Gene Sequence and Phylogenetic Analysis

The genomic DNA of strain KOLBM20 was extracted following the manufacturer’s guidelines, employing the Genomic DNA Isolation Kit (Gene all, Seoul, Republic of Korea). To obtain the 16S rRNA gene, chromosomal DNA from strain KOLBM20 was subjected to amplification utilizing universal bacterial primer sets, 27F/1492R [34]. The complete 16S rRNA gene sequence (1500 bp) was compiled using SeqMan software version 7.1 (DNASTAR Inc., Madison, WI, USA) in conjunction with the BioEdit program [35].

To assess the genetic relatedness between strain KOLBM20 and other *Lactobacillus* species, 16S rRNA gene sequence similarities were retrieved from the GenBank database. Multiple sequence alignments were conducted employing the CLUSTAL X program [36]. The distances between aligned sequences were computed utilizing the Kimura two-parameter method [37]. The construction of a phylogenetic tree was executed using the neighbor-joining method [38] and the maximum-parsimony method [39], facilitated by the MEGA7 Program [40]. In order to establish the robustness of the branches, a bootstrap analysis was performed with 1000 replicates [41].

### 2.3. Preparation of Lactobacillus Iners KOLBM20 Lysate

*Lactobacillus iners* KOLBM20 was cultured in MRS broth at 37 °C for 18 h. Subsequently, the cells were collected through centrifugation, and cellular particles were generated via a high-pressure homogenizer (Avestin Inc., Ottawa, ON, Canada). The resulting lysate was freeze-dried and employed in the experimental procedures. *Lactobacillus iners* KOLBM20 Lysate is lactobacillus ferment lysate under the International Nomenclature Cosmetic Ingredient (INCI) name.

### 2.4. GC-MS Analysis of Extracellular Metabolites

To conduct metabolic profiling on the GC-MS, GC derivatization was carried out. The lyophilized samples were reconstituted in a 100 μL solution of 20 mg/mL methoxyamine hydrochloride in pyridine and then incubated at 37 °C for 1 h to protect carbonyl groups. Subsequently, 100 microliters of N-methyl-N-(trimethylsilyl)-trifluoroacetamide (MSTFA) with 1% trimethyl-chlorosilane (TMCS) was added to each sample, and silylation was performed at 70 °C for 30 min.

Following this, the samples were centrifuged at 14,500× *g* for 15 min, and the resulting supernatant was utilized for GC-MS analysis. One-microliter portions of the samples were introduced into the HP-5MS capillary column (Agilent Technologies, Singapore) using an auto-injector in splitless mode. Helium was employed as the carrier gas at a rate of 1.1 mL/min. The injector temperature and ion source temperature were set at 250 °C and 230 °C, respectively, on the GC-MS instrument (Agilent Technologies, Singapore).

The oven temperature was initially held at 75 °C for 5 min, then increased at a rate of 4 °C per min until reaching a final temperature of 280 °C, which was maintained for 2 min. Data were acquired within the mass-to-charge ratio (*m*/*z*) range of 50 to 500, with a scan time of 0.1 s. Metabolites were identified by referencing the NIST08 mass spectral library and were subsequently normalized using the internal standard ribitol for comparative analysis.

### 2.5. Cell Cultures and Viability Assay

Human skin fibroblasts CCD-986SK cells, Dulbecco’s modified eagle medium (DMEM), and fetal bovine serum (FBS) were acquired from the American Type Culture Collection (ATCC; Manassas, VA, USA). The cells were nurtured in DMEM supplemented with 10% FBS and maintained in a 37 °C incubator under a 5% CO_2_ atmosphere. Cell viability assessments were executed using a CCK-8 (Cell Counting Kit-8), following the manufacturer’s instructions (DOJINDO, Tokyo, Japan).

### 2.6. Ultraviolet Irradiation

The UV light source utilized was a Philips TL 20W/12RS fluorescent sun lamp from Amsterdam, Holland, emitting light in the range of 285 to 350 nm with a peak at 310 to 315 nm. Subsequently, the cells were subjected to a UVB light dosage of 100 mJ/cm^2^.

### 2.7. Cytotoxicity

Cell viability was evaluated through a 3-(4,5-dimethylthiazol-2-yl)-2,5-diphenyltetrazolium bromide (MTT) assay (Sigma, St. Louis, MO, USA). Prior to UVB irradiation, cells were pre-treated with varying concentrations of fucoidan, namely 0, 10, 25, 50, and 100 mg/mL. After incubation periods spanning 24, 48, and 72 h, an MTT solution with a final concentration of 0.5 mg/mL was introduced, and cells were incubated at 37 °C for 3 h. The supernatant was subsequently removed, and 100 mL of dimethyl sulfoxide (DMSO) was added. Ultimately, the absorbance was measured using a microplate reader at 570 nm, enabling the determination of the percentage of viable cells.

### 2.8. Enzyme-Linked Immunosorbent Assay (ELISA) for MMP-1 and Type I Collagen Expression

For assessing MMP-1 and procollagen expression levels, we employed the MMP-1 ELISA kit (R&D systems) and the Procollagen Type I C-Peptide Kit (TaKaRa, Kyoto, Japan). After UVB exposure, CCD-986SK cells were treated with KOLBM20 lysate. The medium obtained following treatment was utilized in the ELISA procedure, following the manufacturer’s instructions.

### 2.9. Western Blotting

Cell lysis was carried out using a lysis buffer composed of the following components: 50 mM Tris–HCl (pH 7.4), 150 mM NaCl, 1 mM EDTA, 1 mM EGTA, 10 mg/mL aprotinin, 10 mg/mL leupeptin, 5 mM phenylme-thanesulfonyluoride (PMSF), and 1 mM dithiothreitol (DTT), containing 1% Triton X-100. After lysis, insoluble debris was removed through centrifugation at 12,000 rpm for 10 min, and the protein content was quantified using Bradford reagent (BioRad, Hercules, CA, USA).

Equal quantities of protein were separated on gradient (10%) SDS PAGE gels (Invitrogen, Carlsbad, CA, USA) and subsequently transferred onto nitrocellulose membranes through electrophoresis. Following this, the membranes were blocked with 5% skimmed milk in TBST (20 mM Tris–HCl, pH 7.6, 137 mM NaCl, 0.05% Tween 20) and incubated with the specified antibodies. The proteins in the Western blots were visualized using enhanced chemiluminescence.

For the Western blotting analysis, the following antibodies were employed: anti-human MMP-1 antibody (1:250) (Calbiochem, San Diego, CA, USA), anti-phospho-Jun N terminal kinase (JNK) (1:250), anti-phospho-extracellular signal-related kinase (ERK) (1:500), anti-total JNK (1:500), and anti-total ERK (1:500) (Cell signaling Boston, MA, USA). In specific experiments, cells were treated with chemical MAPK inhibitors. The MEK inhibitor PD98059 (Calbiochem, San Diego, CA, USA) was added to a final concentration of 10 mM/L, while the JNK inhibitor SP600125 (Calbiochem, San Diego, CA, USA) was added to a final concentration of 25 mM/L for a duration of 30 min.

### 2.10. Statistical Analysis

The presented findings are indicative of a minimum of four distinct experiments and are presented as the mean ± standard error of the mean (SEM). A comparison between the control and treatment groups was conducted through ANOVA variance analysis, followed by a *t*-test for statistical significance assessment. Statistical significance was established when the differences reached a significance level of * *p* < 0.05.

## 3. Results

### 3.1. 16S rRNA Gene Sequence and Phylogenetic Analysis

**Figure 1:**
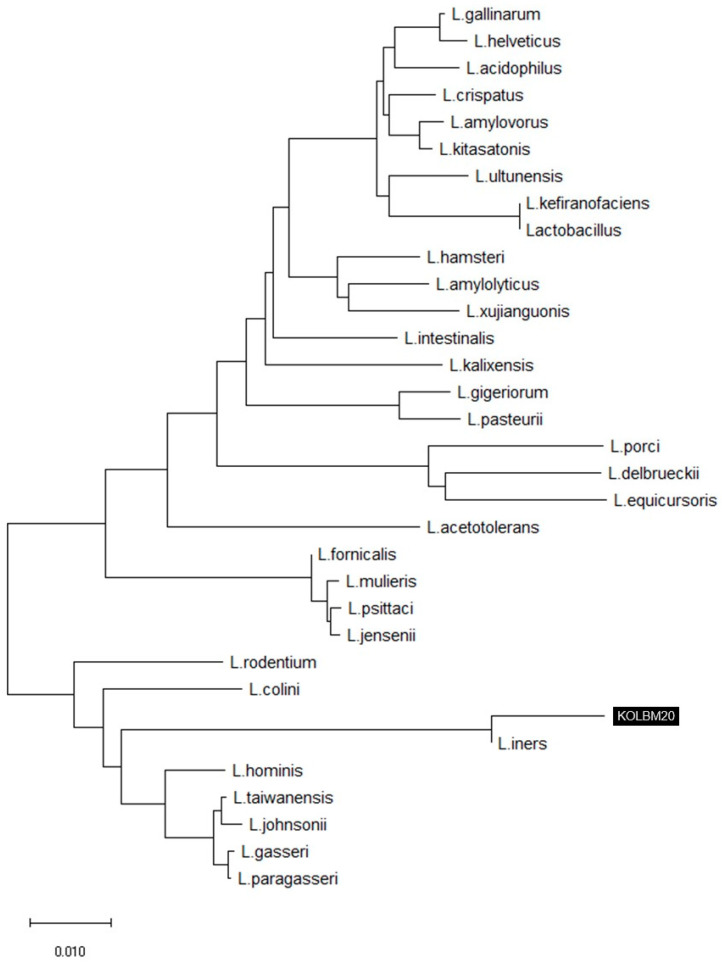
A phylogenetic tree was created using the 16S rRNA gene sequences of *Lactobacillus iners* KOLBM20, which were collected from the skin of women in their twenties. This tree likely illustrates the genetic relationships and diversity among *Lactobacillus iners* strains found on the skin of young women.

The comparative analysis of 16S rRNA gene sequences has revealed that strain KOLBM20 is closely related to the *Lactobacillus* genus, with its closest association being with *L. iners* DSM13335(T), showing a remarkable 99.20% similarity. Furthermore, it exhibits significant similarities to other species, including *L. taiwanensis* DSM21401(T) at 94.04%, *L. johnsonii* ATCC33200(T) at 93.91%, *L. gasseri* ATCC33323(T) at 93.78%, and *L. paragasseri* JCM5343(T) also at 93.78%. Consequently, this strain has been designated as *L. iners* KOLBM20 and is considered a suitable candidate for the specific research focus on the relationship between probiotics and UV. To visualize this genetic relatedness, Figure 1 illustrates the phylogenetic tree constructed using 16S rRNA gene sequences of *L. iners* obtained from the skin of Korean women in their twenties.

### 3.2. Metabolomics Analysis by GC-MS

**Figure 2:**
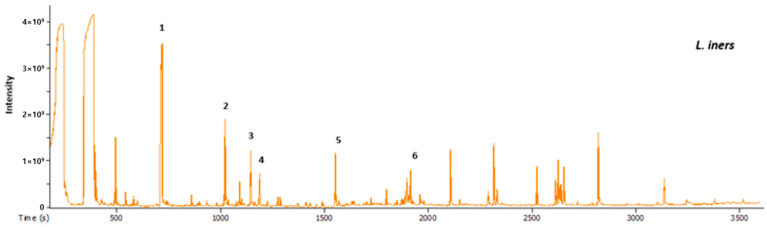
GC-MS metabolomics analysis revealed the presence of six metabolites during fermentation. The most abundant metabolite was Lactic acid (1851.21 mg/L, 1st peak), followed by 2-hydroxyisocaproic acid (HICA) (1335.52 mg/L, 2nd peak), Uracil (971.0 mg/L, 4th peak), Butanedioic acid (685.16 mg/L, 3rd peak), 3-Phenyllactic acid (581.51 mg/L, 5th peak), and Adenine (215.11 mg/L, 6th peak).

In the comparison between human-derived *Lactobacillus* strains and normal *Lactobacillus* strains for use on human skin, the analysis of specific metabolites is essential. A GC-MS analysis, as depicted in Figure 2, revealed six metabolites. Lactic acid, the most abundant at 1851.21 mg/L, is a well-known *Lactobacillus* metabolite with benefits for skin health, including contributing to the skin’s natural acidity and supporting a balanced microbial environment.

The presence of 2-hydroxyisocaproic acid (HICA) at 1335.52 mg/L is intriguing. HICA is identified as a leucine metabolite of Lactobacillus, indicating that certain Lactobacillus strains can produce this compound [42]. While the precise role of HICA in skin health remains unclear, it holds potential benefits when applied to human skin.

Other metabolites identified, such as uracil, butanedioic acid, 3-phenyllactic acid, and adenine, require further investigation to determine their specific functions and effects on human skin. Uracil’s discovery is intriguing because it was found to inhibit UVB-induced wrinkle formation by affecting MMP through NF-κB signaling [43]. In particular, uracil has been reported as a melanogenic inhibitor derived from *L. plantarum* [44]. This information is essential for evaluating the advantages of human-derived Lactobacillus strains over normal strains in skincare applications.

### 3.3. Inhibitory Effect of Strain KOLBM20 Lysate on MMP-1 Secretion and Type I Procollagen Degradation in UVB-Stimulated Dermal Fibroblasts

**Figure 3:**
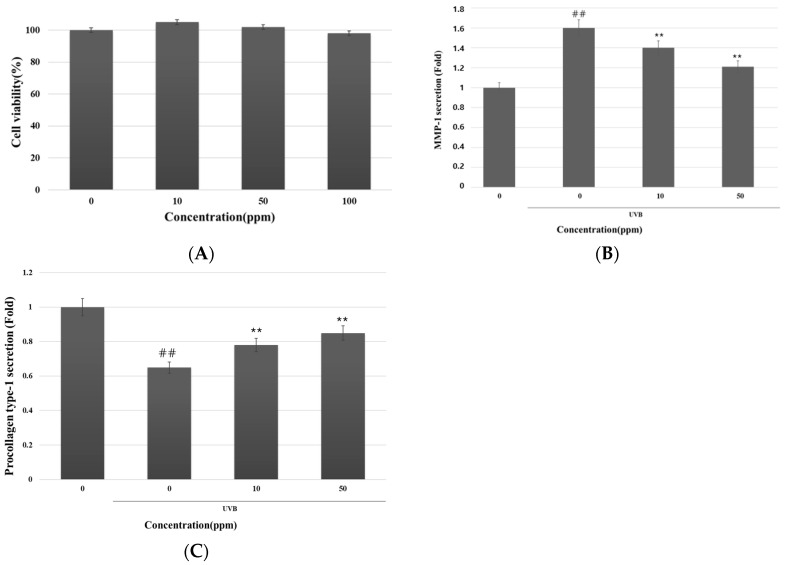
Assessment of cell viability (**A**) and the suppressive influence of KOLBM20 lysate on MMP-1 secretion (**B**) and degradation of type 1 procollagen (**C**) in fibroblast cells stimulated by UVB. The lysate’s inhibitory impact on UVB-induced MMP-1 secretion was further confirmed in human dermal fibroblasts. Significant differences were found when comparing with UV-untreated control: ## *p* < 0.01. Significant differences were found when comparing with UV-treated control: ** *p* < 0.01.

To commence the investigation, we assessed the potential cytotoxicity of strain KOLBM20 lysate on CCD-986SK cells at various concentrations (Figure 3A). The results depicted in Figure 3A clearly show that the strain KOLBM20 lysate did not induce cytotoxic effects within the tested concentration range, extending up to 100 ppm. Despite toxicity assessments being conducted up to 1000 ppm, there was no observable cell death.

Following this, our focus shifted to validating the inhibitory impact of strain KOLBM20 lysate on UVB-induced MMP-1 secretion in CCD-986SK cells. In this experiment, CCD-986SK cells were pre-treated with strain KOLBM20 lysate and then exposed to UVB. The quantification of secreted MMP-1 and type I procollagen levels in the culture medium was carried out using ELISA. Remarkably, strain KOLBM20 lysate exhibited a significant capability to hinder MMP-1 secretion into the culture medium (Figure 3B) and restore the reduced production of type I procollagen (Figure 3C) caused by UVB exposure. This inhibitory effect of strain KOLBM20 lysate on UVB-induced MMP-1 secretion was further confirmed in human dermal fibroblasts. Collectively, these findings strongly suggest that strain KOLBM20 lysate effectively suppresses MMP-1 secretion and mitigates the degradation of type I procollagen in UVB-stimulated dermal fibroblasts.

### 3.4. The MMP-1 Inhibition by Strain KOLBM20 Lysate through the Suppression of Signalling Pathway

**Figure 4:**
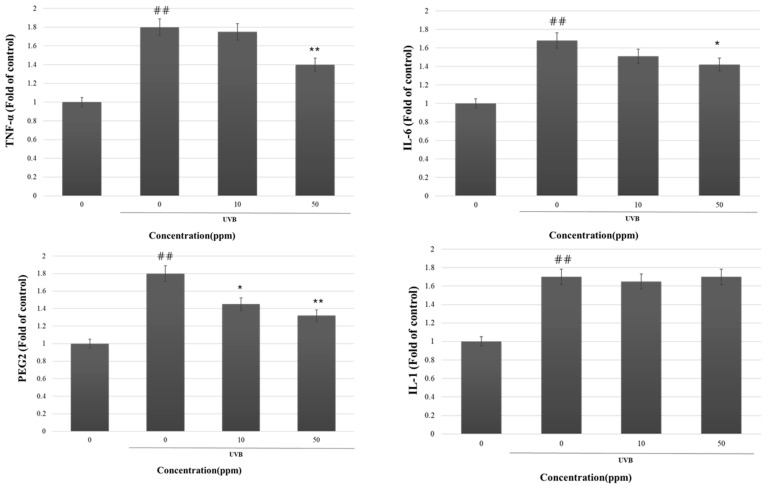
The suppression of MMP-1 secretion by KOLBM20 lysate entailed the inactivation of signaling pathways in fibroblast cells exposed to UVB. Furthermore, KOLBM20 lysate lowered the secretion of TNF-α, IL-1β, IL-6, and PGE2 in HS68 cells following UVB exposure. Significant differences were found when comparing with UV-untreated control: ## *p* < 0.01. Significant differences were found when comparing with UV-treated control: * *p* < 0.05 and ** *p* < 0.01.

Numerous studies have indicated that the synthesis of MMP-1 in skin cells is affected by the mitogen-activated protein kinase (MAPK) pathway in fibroblasts and the ERK signaling pathways in HCS-2/8 cells [45,46]. Managing MMP-1 and MAPK activity presents potential for addressing diverse skin conditions, including aging. With this insight, it was anticipated that the suppressive impact of strain KOLBM20 lysate on MMP-1 secretion might stem from lowered MMP-1 levels. Furthermore, KOLBM20 lysate was observed to diminish the release of pro-inflammatory factors such as TNF-α, IL-1β, IL-6, and PGE2, all induced by UVB in CCD-986SK cells (Figure 4).

This study aimed to understand the molecular mechanism behind the reduction of these inflammatory mediators, including MMP-1, by strain KOLBM20 lysate in UVB-stimulated CCD-986SK cells. Initially, the focus was on the UVB-activated MAPK signaling pathway, as UVB is known to induce MAPK activation in various cell types [47,48]. When CCD-986SK cells were exposed to UVB, the phosphorylation of ERK, p38, and JNK occurred in a time-dependent manner. This phosphorylation peaked at 15 min and gradually decreased.

**Figure 5:**
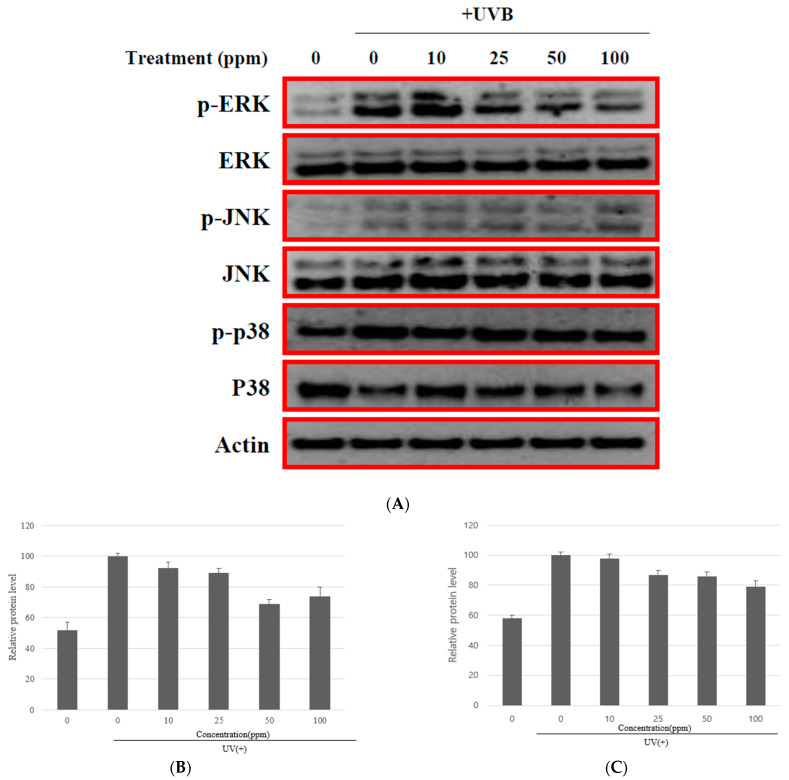
Pre-treatment of CCD-986SK cells with KOLBM20 lysate for 30 min before UVB exposure was found to significantly diminish UVB-induced MAPK phosphorylation. The outcome demonstrated a dose-dependent reduction in the phosphorylation of ERK, p38, and JNK, highlighting the effective action of KOLBM20 lysate (**A**). p-JNK (**B**) and p-p38 (**C**) were visually ambiguous in Western blot, so the density was numerically re-displayed using bar graphs. The experiment was conducted in triplicate.

This study investigated whether strain KOLBM20 lysate could reduce UVB-induced MAPK phosphorylation by pre-treating CCD-986SK cells with the lysate for 30 min before UVB exposure. The results showed that KOLBM20 lysate effectively reduced the phosphorylation of ERK, p38, and JNK in a dose-dependent manner (Figure 5). This suggests that strain KOLBM20 lysate may inhibit the UVB-induced activation of ERK, p38, and JNK, leading to the suppression of MMP-1 expression. The analysis of microbial ingredients revealed the presence of uracil, so it is believed that uracil is responsible for the inhibition of MMP-1 [43].

## 4. Discussion

Strain KOLBM20 was isolated from the facial skin of individuals in their twenties. Comparative analysis of 16S rRNA gene sequences revealed that strain KOLBM20 belongs to the *Lactobacillus* genus and shares its closest genetic affinity with *L. iners* DSM13335(T) (99.20% similarity). To investigate its potential anti-wrinkle activity, we conducted an MMP-1 suppression test. It is well-documented that UV irradiation, both in vitro and in vivo, leads to increased MMP expression, playing a significant role in the premature aging of the skin (photoaging) [49,50]. Recent research has shown a growing interest in the development of compounds from natural sources that can inhibit MMP activity. Our study explored the inhibitory effect of Strain KOLBM20 lysate on MMP-1 as a novel anti-photoaging agent.

While prior studies have reported that *Lactobacillus* species can stimulate dermal fibroblast proliferation and enhance extracellular matrix deposition in vitro, investigations regarding the impact of *Lactobacillus iners*, specifically strain KOLBM20, on MMP-1 expression were lacking. Consequently, we conducted various in vitro experiments to explore the inhibitory effect of *Lactobacillus iners* strain KOLBM20 on MMP-1 expression and elucidated the underlying pathways.

To evaluate the effect of strain KOLBM20 lysate on cell viability following UVB irradiation, cells were pretreated with the lysate. UVB radiation can induce MMP-1 expression, with the extent of damage dependent on the time elapsed after UV exposure. Our findings revealed that strain KOLBM20 lysate, when incubated for 24 h after pretreatment, effectively inhibited UVB-induced MMP-1 expression. This inhibitory effect persisted after 48 and 72 h of incubation. Metabolite analysis through GC/MS indicated a significant presence of uracil in KOLBM20. Previous research has suggested that uracil has the capacity to inhibit MMP-1 protein and mRNA expression, aligning with our results. Consequently, strain KOLBM20 lysate may mitigate connective tissue damage by inhibiting UVB-induced MMP-1 expression, with uracil potentially playing a role in this process by modulating the immune pathway.

This study focused on the impact of strain KOLBM20 lysate on reducing pro-inflammatory factors (TNF-α, IL-1β, IL-6, and PGE2) induced by UVB. Exposure to UVB induces the production of IL-1β, IL-6, and TNF-α by human skin cells [51]. Additionally, it generates PGE2 [52], most of which are cytokines associated with inflammation. The inflammatory mediators mentioned can affect the structural integrity of the skin, contributing to the development of wrinkles. Inhibiting these cytokines is crucial in the early suppression of skin inflammation, as inflammation is a fundamental cause of skin aging [53], and caring for cytokines related to inflammation is highly important. The lysate of strain KOLBM20 addresses crucial inflammation in photoaging by suppressing UVB-induced TNF-α, IL-1β, IL-6, and PGE2.

A key aim was to clarify the molecular mechanism responsible for diminishing inflammatory mediators, particularly MMP-1, in cells stimulated by UVB. This study initially concentrated on the UVB-activated MAPK signaling pathway, recognizing the role of UVB in initiating MAPK activation across various cell types [47,48]. In our study, we observed the involvement of ERK and JNK signaling pathways in UVB-induced MMP-1 expression. Similar to previous research, we noted the activation of ERK and JNK kinases within 30 min of UVB irradiation, followed by a return to baseline levels in cells. The inhibitory effect of strain KOLBM20 lysate on UVB-induced MMP-1 expression was associated with the suppression of these pathways. Consequently, we hypothesize that strain KOLBM20 lysate’s inhibition of MMP-1 expression is linked to the prevention of the ERK and JNK signaling pathway activation. Given previous reports on uracil’s role in regulating ERK, JNK, and p38, it is plausible that the lysate’s MMP-1 inhibition is mediated, at least in part, by uracil [43].

In summary, our study demonstrates that strain KOLBM20 lysate exerts an inhibitory effect on MMP-1 expression at the transcriptional level. Additionally, our data indicate that strain KOLBM20 lysate may prevent UVB-induced MMP-1 expression by suppressing the UVB-induced activation of the ERK, JNK, and p38 signaling pathways. Therefore, strain KOLBM20 lysate holds promise as a potential therapeutic agent for the prevention and treatment of skin photoaging.

## 5. Conclusions

In this study, we were able to confirm the following facts:

1. Strain KOLBM20 was identified as a member of the *Lactobacillus* genus and found to have a close genetic affinity with *L. iners* DSM13335(T), sharing a 99.20% similarity in 16S rRNA gene sequences.
2. We also confirmed the anti-aging properties of strain KOLBM20 lysate, specifically its potential to inhibit MMP-1 expression.
3. While previous research has shown that *Lactobacillus* species can stimulate dermal fibroblast proliferation and enhance extracellular matrix deposition in vitro, the specific impact of *Lactobacillus iners* strain KOLBM20 on MMP-1 expression had not been previously explored.
4. Our research revealed that strain KOLBM20 lysate effectively reduced the expression of MMP-1 induced by UVB irradiation in in vitro experiments. This inhibitory effect persisted over time, possibly due to the presence of uracil in KOLBM20, a compound known to inhibit MMP-1 expression.
5. We identified the involvement of the ERK and JNK signaling pathways in UVB-induced MMP-1 expression. Strain KOLBM20 lysate was found to suppress these pathways, suggesting a potential mechanism for its inhibition of MMP-1 expression.

In summary, our study concludes that *L. iners* KOLBM20 lysate shows promise as a novel therapeutic agent for the prevention and treatment of skin photoaging. This potential is based on its ability to inhibit MMP-1 expression and suppress specific signaling pathways activated by UVB radiation. Overall, our research suggests that strain KOLBM20 lysate, derived from a *Lactobacillus* bacterium, has the potential to be an effective intervention in addressing the effects of UV radiation on the skin’s aging process.

## Data Availability

The data that support the findings of this study are available from the corresponding author upon reasonable request.
